# Non‐Toxic Tau Oligomer Polymorph Isolated from Non‐Demented Individuals with Alzheimer's Neuropathology Brains

**DOI:** 10.1002/alz70855_103856

**Published:** 2025-12-24

**Authors:** Jutatip Guptarak, Batbayar Tumurbaatar, Michela Marcatti, Shrinath Kadamangudi, Pietro Scaduto, Wenru Zhang, Adam Trupp, Maria‐Adelaide Micci, Giulio Taglialatela, Anna Fracassi

**Affiliations:** ^1^ University of Texas Medical Branch, Galveston, TX, USA

## Abstract

**Background:**

Alzheimer's disease (AD) is the most prevalent neurodegenerative disorder and the leading cause of dementia worldwide. Tau oligomers (tauO) are among the most toxic molecular species implicated in AD, making them a key focus for diagnostic and therapeutic strategies. Intriguingly, neuropathological autopsy studies reveal that approximately one‐third of individuals with extensive AD pathology—marked by neurofibrillary tangles (NFTs) and amyloid plaques—exhibit no clinical signs of dementia. These individuals are classified as Non‐Demented with Alzheimer's Neuropathology (NDAN). NDAN individuals exhibit resilience to cognitive decline, suggesting that tauO in these cases might propagate in a non‐toxic form without inducing neuronal death.

**Method:**

Brain‐derived tau oligomers (BDTO) were isolated from both AD patients and NDAN individuals through immunoprecipitation, followed by biochemical and biophysical characterization. Coupled with LTP and cell survival assays, the analyses revealed distinct conformations of AD and NDAN BDTO. NDAN BDTO exhibited significantly reduced synaptic and neuronal toxicity ex vivo and *in vivo* compared to AD BDTO. Intracerebroventricular (ICV) injections of AD‐BDTO and NDAN‐BDTO into wild‐type mice were performed to examine their effects on key cellular pathways.

**Result:**

Mice injected with NDAN‐BDTO displayed preserved autophagy, as evidenced by increased levels of autophagy markers such as Beclin‐1, Atgs, LC3‐II, indicating an active autophagic response. Furthermore, these mice demonstrated a robust antioxidant response, with elevated expression of enzymes like superoxide dismutase (SOD1, SOD2) and catalase, coupled with reduced oxidative damage, as indicated by lower levels of 8‐oxo‐dG. Additionally, NDAN‐BDTO‐injected mice exhibited highly phagocytic microglia as evidenced by increased expression of markers associated with phagocytosis, such as CD68. Behavioral assessments, including the novel object recognition (NOR) test, showed significantly better memory retention in NDAN‐BDTO‐injected mice compared to their AD‐BDTO counterparts, aligning with the molecular resilience observed.

**Conclusion:**

Our findings suggest that NDAN brains harbor a non‐toxic tauO capable of propagating without inducing neuronal death or cognitive decline. These insights open new translational avenues, proposing that converting tauO into less toxic forms could effectively halt AD progression. Moreover, the possibility that tau in NDAN individuals carries mutations or structural modifications that render it less toxic is under investigation, opening new avenues for understanding resilience mechanisms in AD

